# Zootechnical and reproductive performance of juvenile male *Astyanax lacustris* (Yellowtail Lambari) cultivated in Biofloc Technology (BFT) and Recirculating Aquaculture System (RAS)

**DOI:** 10.1590/1984-3143-AR2025-0015

**Published:** 2025-10-17

**Authors:** Jéssica Julian Fernandes Lima, Maiara Luzia Grigoli Olivio, Lorena Pacheco da Silva, Malbelys Padilla Sanchez, Stella Indira Rocha Lobato, Lais Pedroso Borges, Laicia Carneiro-Leite, Luciane Gomes da Silva, Ruy Alberto Caetano Corrêa, Hellen Buzollo, Rosicleire Veríssimo-Silveira, Alexandre Ninhaus-Silveira

**Affiliations:** 1 Programa de Pós-graduação em Ciência e Tecnologia Animal, Universidade Estadual Paulista “Júlio de Mesquita Filho” – Unesp, Ilha Solteira, SP, Brasil; 2 Programa de Pós-graduação em Ciências Biológicas (Biologia), Universidade Estadual Paulista “Júlio de Mesquita Filho – Unesp, Botucatu, SP, Brasil; 3 Universidade Federal de Mato Grosso do Sul – UFMS, Campo Grande, MS, Brasil; 4 Centro Universitário de Rio Preto – UNIRP, São José do Rio Preto, SP, Brasil; 5 Laboratório de Ictiologia Neotropical – LINEO, Departamento de Biologia e Zootecnia, Universidade Estadual Paulista “Júlio de Mesquita Filho” – Unesp, Ilha Solteira, SP, Brasil

**Keywords:** fish reproduction, seminal traits, sperm kinetics, sustainable aquaculture

## Abstract

A total of 1600 juvenile *Astyanax lacustris* (commonly known as yellowtail lambari) with an initial age of two months were used. Fish were subjected to two systems: biofloc technology (BFT) and clear water recirculation (RAS) in a completely randomized design. Replicates were established for each treatment, and carbon sources and carbon ratios were adjusted specifically for BFT tanks to optimize microbial floc formation. Feeding was based on 3% of the total biomass of each tank, which was reduced to 1% when the fish reached four months of age. The gonadal factor and gonadosomatic index (IGS) were superior in fish cultured in the RAS system during the third month of culture, although all gonads from both BFT and RAS systems showed reproductive capability based on histological analysis. The hepatosomatic index (IHS) was higher in the BFT system in the third month. BFT males exhibited a higher percentage of dry matter and ether extract in body composition, while RAS males had a higher percentage of crude protein and ash. At five months, RAS males displayed superior total progressive motility, rapid sperm count, and flagellar beat frequency compared to BFT males. By fourteen months, RAS males had sperm with higher total motility, VSL (curvilinear velocity), VSL (linear velocity), and VAP (average trajectory velocity) than BFT males. Based on these results, BFT proves effective for the general cultivation and reproductive maintenance of *Astyanax lacustris*, although RAS offers slight advantages in seminal quality for male fish.

## Introduction

Biofloc technology (BFT) is an innovative aquaculture system that relies on the transformation of nitrogenous compounds dissolved in water by introducing carbon sources, which promote the formation of microbial biomass. This biomass, in turn, acts as a natural food source rich in proteins, fatty acids, vitamins, and other essential nutrients, benefiting the growth and health of farmed species (Khanjani et al., 2023a; Fóes et al., 2012). BFT systems provide significant advantages in terms of productivity, reduced water usage, enhanced biosecurity, and decreased reliance on high-protein feed, making them a sustainable choice for aquaculture, particularly in shrimp farming (Wasielesky, 2024; Khanjani et al., 2023b). Furthermore, BFT systems support the cultivation of species in areas distant from coastal regions, expanding their applicability.

Recirculating Aquaculture Systems (RAS), on the other hand, are closed-loop systems that use advanced filtration and water treatment technologies to recycle water within the culture environment. This approach reduces water consumption, enables precise control over water quality parameters, and minimizes the discharge of waste into surrounding ecosystems. RAS is widely used for species that require controlled water conditions for optimal growth and production, such as trout, salmon, and tilapia, and has proven to be effective for commercially valuable species due to its high biosecurity and suitability for high-density farming (Khanjani et al., 2024a). In this study, we selected RAS as a comparative system to BFT to evaluate its efficiency and sustainability, particularly focusing on the effects of water quality management and cost-effectiveness for farming *Astyanax lacustris*. Comparing BFT and RAS in this context provides valuable insights into which system better supports the zootechnical and reproductive development of this species under controlled conditions.

*Astyanax lacustris*, a small Neotropical fish species, holds potential for commercial aquaculture due to its omnivorous feeding behavior, adaptability to diverse culture conditions, and ability to reproduce throughout much of the year (Nomura, 1975; Agostinho et al., 1984; Evangelista et al., 2019). This species was chosen for the study because it meets the biological and environmental requirements compatible with BFT, such as high tolerance for nutrient-dense water and an omnivorous diet, which aligns well with the microbial composition of bioflocs. Additionally, *A. lacustris* is used for human consumption, as live bait in sport fishing, and as a source of fish meal, making it an economically promising candidate for cultivation in biofloc systems. Although the species' growth and reproductive performance in BFT are underexplored, its potential has been demonstrated in other high-density systems.

From a biological perspective, *A. lacustris* reaches sexual maturity relatively early, with optimal temperature conditions between 24-28 °C supporting gonadal development, spawning, and larval growth (Hayashi et al., 2004; Salaro et al., 2008). Its feeding behavior and nutritional requirements, particularly protein needs, are suited to the high-protein content bioflocs can offer. These characteristics support the rationale for selecting *A. lacustris* for evaluation in BFT, as the technology could provide continuous nutrient availability and stable water quality, promoting reproductive success and sustainable growth.

This study aims to evaluate and compare the zootechnical and reproductive development of male *A. lacustris* cultivated in BFT and RAS, contributing unprecedented data on the viability of these systems for farming this Neotropical species and shedding light on the broader potential of biofloc-based aquaculture.

## Methods

### Location and animals

For this study, juveniles of Astyanax lacustris were used, originating from the breeding stock at the Laboratory of Neotropical Ichthyology (L.I.NEO), Universidade Estadual Paulista “Júlio de Mesquita Filho,” Ilha Solteira Campus, where the experiment was conducted according to the standards approved by the Ethics Committee for the Use of Animals in Scientific Experiments (CEUA-FEIS/UNESP 02/2021).

### Experimental design

The experiment lasted one year (365 days), with an initial base of 1600 *Astyanax lacustris* juveniles, distributed across two aquaculture systems: biofloc technology (BFT) and a recirculation aquaculture system (RAS). A conventional rearing system was included as a control to serve as a reference for traditional farming methods.

Each aquaculture system comprised four 1000 L tanks, totaling four replicates per treatment. The temperature was set to 27 ºC in all tanks, based on studies that demonstrate this as the optimal temperature for growth and metabolic stability in tropical species like *Astyanax lacustris* (Schleger et al., 2022). This temperature control helps improve feed conversion and growth parameters, providing conditions that meet the physiological needs of the species while avoiding oxidative stress in controlled environments.

In the BFT system, an initial 1 kg of NaCl was added to each tank at the start of the experiment to help reduce the toxicity of nitrogen compounds, particularly nitrite, which is common in biofloc systems. Adding NaCl improves fish osmoregulation and reduces nitrite toxicity by competing with nitrite for absorption sites on the fish's gills, as noted in studies by Wang et al. (2018).

For the biofloc culture, two 1000 L tanks were pre-inoculated prior to the experiment’s onset. Each experimental tank received 100 L of pre-formed bioflocs (10% of the tank volume). The biofloc culture was maintained with weekly adjustments to the carbon ratio (12:1) using powdered sugarcane molasses as a carbon source, following standard procedures for optimizing suspended solids. Weekly measurements of settleable suspended solids (SS) were taken in the morning using a 1000 ml Imhoff cone, quantifying SS in ml.L^−1^ after one hour of sedimentation.

### Feed management and analysis of water parameters

The animals were fed twice a day (09:00 a.m. and 05:00 p.m.) with commercial feed containing 32% CP. Initially, feed was provided at 3% of total biomass, adjusted to 1% by the fourth month when the fish reached sexual maturity (Porto-Foresti et al., 2010). Biometric measurements were taken monthly on 20% of the fish in each tank to adjust feed accurately.

Water quality parameters, including dissolved oxygen (DO), temperature (ºC), and pH, were measured twice daily (09:00 a.m. and 05:00 p.m.) using a multiparameter analyzer (AKSO – AK88). Concentrations of ammonia, nitrite, and nitrate were tested thrice weekly using Labcon Test kits. At the beginning of the experiment, 1 kg of NaCl was added to each BFT tank to control nitrite.

### Sampling and variables analyzed

Zootechnical performance, gonadosomatic and hepatosomatic indices, condition factors, gonadal histology, and seminal analysis were assessed monthly for the initial three months of the experiment, with only seminal analysis repeated after one year.

### Zootechnical performance

During the first three months, 20% of the fish in each tank were weighed monthly and measured for total and standard lengths. Biomass gain, specific growth rate, apparent feed conversion, and protein efficiency rate were calculated. Specific parameters include Total Biomass Gain (GBT), Specific Growth Rate (SGR), Survival Rate, Apparent Feed Conversion (AFC), and Protein Efficiency Rate (PER).

### Proximate analysis of bioflocs and fish body composition

Samples of bioflocs and fish (viscera included) were taken at the beginning and after the third month. Samples were stored in plastic containers at -20 °C and analyzed for dry matter, ash, crude protein (Kjeldahl method), and fat (Soxhlet method), based on AOAC (2000) guidelines.

### Gonadosomatic, hepatosomatic indices, and condition factors

Monthly, three males were sampled from each tank for liver and gonadal mass measurements. Metrics were calculated as follows: Condition Factor (K), Gonadal Condition Factor (ΔK), Hepatosomatic Index (HSI), and Gonadosomatic Index (GSI), following Vazzoler (1996) and Vazzoler et al. (1989) guidelines.

### Histological analysis of gonads

Gonads were fixed in 2.5% glutaraldehyde, processed in historesin glycol methacrylate (LEICA®), sectioned at 3.0 µm, and stained with Hematoxylin and Eosin. The reproductive cycle was classified according to Brown-Peterson et al. (2011).

### Reproductive character analysis

Semen was collected at five and 14 months using hormonal induction with carp pituitary extract (3 mg/kg), administered as a single dose, with collection via abdominal massage. The C.A.S.A. system was used to analyze sperm motility.

#### Kinetic aspects of sperm motility

The C.A.S.A. system was used for sperm motility analysis. (ISAS® Integrated Semen Analysis System, Proiser, Valencia, Spain), consisting of a computer coupled to an analog camera (ISAS 782C, Proiser, Spain) connected to a microscope (UB200i (UOP / Proiser) with a phase contrast objective negative 10x. The software was calibrated to capture 25 images/second (FPS), considering a minimum sperm velocity of 10µm/s. The kinetic parameters evaluated were Total motility (MT), progressive motility (MP), curvilinear velocity (VCL, μm/s), linear velocity (VSL, μm/s), average velocity (VAP, μm/s), percentage of fast, medium and slow sperm, linearity coefficient (LIN, %), straightness coefficient (STR, %), mean oscillation of the spatial trajectory of the sperm (WOB, %), amplitude of lateral movement of the head (ALH, μm) and flagellar beat frequency (BCF, Hz). For seminal analysis, the semen was placed in a Chamber from Makler (Sefi – Medicals Instruments), being activated with distilled water in a ratio of 1:1000 µl (semen: activator).

### Sperm concentration (sperm/mm^3^)

To estimate the sperm concentration, a Neubauer Improved hematimetric chamber (bright line; HBG) was used. The semen was collected from three specimens per tank and diluted in a formaldehyde-saline solution at a ratio of 1:1000 µl (semen: solution), following the methodology recommended by Ninhaus-Silveira et al. (2002).

### Statistical analyzes

The dependent variables were subjected to the Shapiro-Wilk test for normality and Levene’s test for homogeneity of variances. Initial weight (g) was used as a covariate to analyze the following parameters: final weight (g), weight gain (g), daily weight gain (g), and apparent feed conversion. Initial biomass was used as a covariate to analyze the total biomass gain (TBG) (kg). After verifying normality and homogeneity of variances, the dependent variables were analyzed using a model with one independent variable (one-way ANOVA). ANOVA was used to evaluate global differences among the treatments, while the t-test was applied for direct pairwise comparisons when appropriate. Each tank was considered an experimental unit, and each treatment consisted of four tanks. All analyses were performed following Zar (2010) and using the Statistical Analysis System (SAS, 2002). The significance level adopted for all tests was 0.05.

## Results

### Water quality

The parameters of dissolved oxygen, temperature, and pH did not differ (p>0.05) between BFT and RAS cultures (Table 1). However, the standard deviation for temperature was relatively high in both systems, suggesting potential variation among tanks or over the months. This variability could have influenced the dependent variables, and future studies should address these fluctuations in more detail to ensure comprehensive reporting of environmental conditions.

**Table 1:** Mean values (±standard deviation) of water quality parameters of lambari-de-rabo-amarelo *Astyanax lacustris* in biofloc tecnology and recirculation during three months.

**SYSTEM**	**PARAMETERS**		
	**Dissolved oxygen (mg/L)**	**Temperature (ºC)**	**pH**
BFT	6.59±1.07	25.92±2.39	8.64±0.28
RAS	6.74±0.93	26.47±2.27	8.65±0.33

**Note:** BFT = Biofloc Tecnology; RAS = Recirculation System.

In the biofloc system, the mean volume of settleable solids was 9.94 ml/L. During the first month, when it was necessary to add a carbon source, the values reached 20.5 mL/L. From the second month onward, these values decreased, stabilizing at 6.75 mL/L.

In the first experimental month, ammonia (NH_3_) in the RAS ranged from 0 to 0.5 mg/L. This value later dropped to zero and remained stable. In contrast, ammonia in the BFT system peaked in the first month at 3.5 mg/L, then gradually decreased, stabilizing at 1.0 mg/L. Nitrite (NO_2_^−^) in the RAS ranged from 0 to 0.5 mg/L throughout the three months, while in the BFT system, it peaked in the second month, reaching 2.2 mg/L before decreasing at the beginning of the third month. Nitrate (NO_3_^−^) values were more comparable between systems, peaking in the second month at 100 mg/L in BFT and 50 mg/L in RAS. In the third month, nitrate levels decreased to maximum values of 2 mg/L in BFT and 25 mg/L in RAS.

### Zootechnical performance

After three months of cultivation, the total biomass gain did not differ (p>0.05) between the BFT (5.23 ± 0.24 kg) and RAS (5.37 ± 0.68 kg) systems. Similarly, feed conversion, specific growth rate, protein efficiency rate, and condition factors (both with and without gonad influence) did not show significant differences (p>0.05) between the two systems. However, the hepatosomatic index was higher (p = 0.007) in fish produced in BFT (0.90 ± 0.21%) than in fish produced in RAS (0.64 ± 0.21%) (Table 2).

**Table 2:** Biological parameters (mean±standart deviation) of lambari-do-rabo-amarelo *Astyanax lacustris* (five months of age) after three months of cultivation under biofloc technology and recirculation with clear water.

**Zootechnical indexes**	**Systems**		**P-value**	**CV (%)**
	**BFT RAS**			
**TBG (kg)**	5.23±0.24	5.37±0.68	0.575	6.13
**AFC**	1.92±0.08	1.98±0.32	0.666	9.62
**SGR (%)**	1.28±0.04	1.30±0.04	0.636	3.31
**PER (%)**	0.86±0.03	0.83±0.14	0.895	11.12
**HI (%)**	0.90±0.21^a^	0.64±0.21^b^	0.007	12.27
**K**	1.17±0.14	1.22±0.06	0.14	3.51
**K’**	1.14±0.14	1.19±0.06	0.18	3.59
**S (%)**	100	100	-	-

**Legends:** BFT = Biofloc Tecnology; RAS = Recirculation System; CV = Coefficient os Variation; TBG = Total Biomass Gain; AFC = Apparent Feed Conversion; SGR = Specific Growth Rate; PER = Protein Efficiency Rate; HI = Hepatosomatic Index; K = Condition Factor Under Gonad Influence; K’ = Condition Factor Without Gonad Influence; S = Survival. Medians on the same line differ statistically from each other when P < 0.05.

### Analysis of the centesimal composition of the biofloc

Bioflocs from the four cultivation tanks showed mean values of 6.65% dry matter, 45.71% ash, 0.61% ether extract, and 20.36% crude protein (Table 3). No statistical significance tests were reported for the proximate composition data; however, average values are provided for comparative purposes.

**Table 3:** Bromatological analisys of bioflocs.

**Tank**	**Components**			
	**DM (%)**	**AS (%)**	**EE (%)**	**CP (%)**
**1**	6.03	45.63	0.62	20.45
**2**	7.13	46.55	0.45	20.72
**3**	7.37	47.36	0.62	19.35
**4**	6.05	43.29	0.73	20.93

Note: DM = Dry Matter; AS = Ashes; EE = Ether Extract; CP = Crude Protein.

Dry matter and ether extract were higher in fish produced in BFT than in RAS, while ash and protein content were higher in fish produced in RAS (Table 4).

**Table 4:** Body bromatological analysis of *Astyanax lacustris* males grown in biofloc and recirculation technology (mean±standart deviation).

**System**	**Components**			
	**DM (%)**	**AS (%)**	**EE (%)**	**CP (%)**
**BFT**	34.05±0.09^a^	12.84±0.61^b^	39.09±2.20^a^	49.68±1.94^b^
**RAS**	30.88±0.47^b^	14.69±0.10^a^	30.88±2.45^b^	53.35±1.63^a^
*P-Value*	0.001	0.006	0.0004	0.025
*CV (%)*	1.75	4.30	7.50	4.25

Legends: BFT = Biofloc Tecnology; RAS = Recirculation System; CV = Variation Coefficient; DM = Dry Matter; AS = Ashes; EE = Ether Extract; CP = Crude Protein. Differents letters in the same column differ statistically from each other (P<0.05).

### Gonadosomatic index and gonadal condition factor

**Figure 1:**
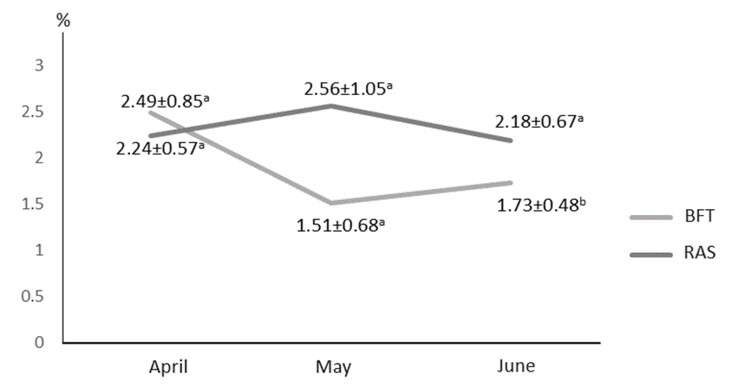
Gonadasomatic index (%) during the experimental period.

No statistical difference (p>0.05) was found in gonadal condition factors between the two systems. However, the gonadosomatic index was higher in the RAS system by the end of the third month of cultivation (Figure 1).

### Histological analysis of the gonads

All fish whose gonads were analyzed were deemed reproductively suitable, indicating that both culture systems allowed the reproductive cycle to proceed and sperm cells to develop appropriately.

### Seminal quality

At the end of the third month, A. lacustris cultivated in RAS showed higher values for sperm kinetic parameters PROG, fast SPTZ, and BCF. There were no significant differences detected for MOT, medium and slow SPTZ, VCL, VSL, VAP, LIN, STR, WOB, ALH, and CONC between treatments (Table 5). After one year of cultivation, fourteen-month-old males in the RAS system exhibited significantly higher values for MOT, VCL, VSL, and VAP, whereas only mean SPTZ was higher in BFT (Table 6).

**Table 5:** Parameters of seminal quality of lambari-do-rabo-amarelo (five months of age), in the third month of cultivation in BFT and RAS (mean±standart deviation).

**Sperm characteristics**	**System**		***P- Value***	***CV***
	**RAS**	**BFT**		
**MOT (%)**	75.45±0.19	57.08±0.11	0.061	16.29
**PROG (%)**	56.97±0.17^a^	40.68±0.09^b^	0.048	18.09
**FSPTZ (%)**	53.72±0.17^a^	33.91±0.14^b^	0.042	23.52
**MSPTZ (%)**	15.51±0.07	15.44±0.05	0.977	21.02
**SSPTZ (%)**	6.21±0.02	7.72±0.02	0.138	17.41
**VCL (µm/s)**	59.25±7.29	51.02±7.00	0.065	8.93
**VSL (µm/s)**	49.82±6.77	43.22±5.58	0.083	9.30
**VAP (µm/s)**	55.98±7.57	48.48±6.00	0.079	9.21
**LIN (%)**	84.71±2.90	83.70±3.47	0.425	1.95
**STR (%)**	88.83±2.06	88.30±2.63	0.567	1.38
**WOB (%)**	95.30±1.39	94.72±1.28	0.322	0.79
**ALH (µm)**	1.21±0.04	1.20±0.05	0.588	2.54
**BCF (Hz)**	7.49±0.22^a^	7.36±0.17^b^	0.010	0.63
**CONC** (x10^9^ sperm/mL)	8.16±3.07	10.28±1.67	0.150	19.09

**Legends:** BFT = Biofloc Tecnology; RAS = Recirculation System; CV = Coefficient os Variation; MOT = Total Motility; PROG = Progressivity; FSPTZ = Fast Spermatozoa; MSPTZ = Medium Spermatozoa; SSPTZ = Slow Spermatozoa; VCL = Curvilinear Velocity; VSL = Linear Velocity; VAP = Avarage Trajectory Velocity; LIN = Linearity Coefficient; STR = Rectilinearity Coefficient; WOB = Mean Oscillation of the Sperm Spatial Trajectory; ALH = Lateral Range of Motion of the Head; BCF = Flagellar Beating Frequency; CONC = Sperm Concentration. Different letters indicate statistical difference in the same row (P<0.05).

**Table 6:** Parameters of seminal quality of lambari-do-rabo-amarelo (fourteen months of age), in the tenth month of cultivation in BFT and RAS (mean±standart deviation).

**Sperm characteristics**	**System**			***P-Value***	***CV***
	**RAS**	**BFT**			
**MOT (%)**	85.95±0.07^a^		80.4±0.04^b^	0.031	7.05
**PROG (%)**	68.1±0.07	61.0±0.05		0.096	10.20
**FSPTZ (%)**	72.15±0.23	57.90±0.09		0.198	28.18
**MSPTZ (%)**	8.85±0.22^b^	14.55±0.06^a^		0.034	131.89
**SSPTZ (%)**	4.80±0.02	4.55±0.02		0.212	41.63
**VCL (µm/s)**	75.05±13.02^a^	63.0±5.52^b^		0.028	15.71
**VSL (µm/s)**	62.70±12.76^a^	52.80±4.79^b^		0.023	17.93
**VAP (µm/s)**	69.90±13.35^a^	58.80±5.57^b^		0.049	17.14
**LIN (%)**	85.25±4.21	82.80±2.37		0.325	3.96
**STR (%)**	89.95±2.06	88.35±2.63		0.364	4.30
**WOB (%)**	94.70±3.20	94.10±0.79		0.344	2.42
**ALH (µm)**	1.10±0.03	1.10±0.00		0.449	1.82
**BCF (Hz)**	14.5±0.82	14.21±0.55		0.371	4.81
**CONC (x10^9^ sperm/mL)**	12.90±4.75	10.93±5.12		0.546	38.29

**Legends:** BFT = Biofloc Tecnology; RAS = Recirculation System; CV = Coefficient os Variation; MOT = Total Motility; PROG = Progressivity; FSPTZ = Fast Spermatozoa; MSPTZ = Medium Spermatozoa; SSPTZ = Slow Spermatozoa; VCL = Curvilinear Velocity; VSL = Linear Velocity; VAP = Avarage Trajectory Velocity; LIN = Linearity Coefficient; STR = Rectilinearity Coefficient; WOB = Mean Oscillation of the Sperm Spatial Trajectory; ALH = Lateral Range of Motion of the Head; BCF = Flagellar Beating Frequency; CONC = Sperm Concentration Value x10^9. Medians on the same line differ statistically from each other when P<0.05.

## Discussion

Conditions with and without gonad influence are good indicators of fish welfare (Agostinho et al., 1990), and the fact that there was no difference in this indicator between animals cultured in the two systems tested, together with the fact that although there was no mortality, it can be considered that the two systems tested for the cultivation of *Astyanax lacustris* were efficient in maintaining adequate environmental and nutritional parameters for the development of the species.

Regarding BFT and the concentration of bioflocs, Emerenciano et al. (2017) indicated that the level of settleable solids required for raising *Oreochromis niloticus* fingerlings must be between 5 and 20 ml/L-1. Thus, considering that the average level of bioflocs maintained in this experiment was within this range, and that data relating to the zootechnical development of *A. lacustris* indicated that the level of solids was also suitable for the species, it can be considered that this concentration of bioflocs is ideal for freshwater fish species with omnivorous eating habits.

Omnivorous fish have a lower protein requirement because they have a longer intestine, with food remaining in contact with enzymes longer (Booth, Moses, and Allan, 2013). Because fish species of the genus *Astyanax* are categorized as omnivores, there is no need to provide food with high protein value for their development (Cotan et al., 2006). Massago and Silva (2020) studied protein levels (26.5%, 28.6%, and 31.2% of CP) in *Astyanax bimaculatus*, where the fish developed similarly for all indices used to measure zootechnical productivity. Higher hepatosomatic index values for animals in the BFT system were expected because the high amount of protein (feed + biofloc biomass) available in the environment may have caused an increase in liver weight, as this organ is one of the main deamination sites for amino acids (Bombardelli et al., 2003; Khanjani et al., 2024b).

Cyrino et al. (2000) demonstrated that diets with an average crude protein content of 42% caused an increase in the accumulation of hepatic glycogen and an increase in the hepatosomatic index in juveniles of Black Bass *Micropterus salmoides*, as well as in a study on *O. niloticus* (Abdel-Tawwab et al., 2010), in which excess protein in the diet caused an accumulation of energy in the animals' bodies in the form of glucose, being stored as glycogen in the liver, increasing the liposome index in the fish, which ultimately increased the visceral fat rate in animals. These data corroborate the data obtained in this experiment, in which the highest hepatosomatic indices were observed in fish exposed to BFT, which also presented a higher percentage of ether extract in their body composition, which is certainly related to the available food, as they had constant access to biofloc particles, without consumption control.

In females, IHS values are related to the reserve and mobilization of proteins and lipids, which will be part of the growth of oocytes during the gonadal development process, thus resulting in an increase in IGS concomitantly with a decrease in IHS, with the process of gonadal maturation (Sayer et al., 1995). However, for males, Barreto et al. (1998) and Cardoso et al. (2013) indicated that this inverse correlation between IHS and IGS should not be considered, as in reproductive periods, the IHS and IGS trends are similar.

The lower percentage of protein and higher percentage of ether extract in the body composition of male *Astyanax lacustris* cultivated at BFT can be explained by the animals having biofloc availability in the environment throughout the experimental period (average protein value of 20.36%), in addition to the artificial food (ration with 32% CP), since food levels above demand provide excess energy (ether extract) in the diet and which may have caused the accumulation of visceral and muscular fat (NRC, 1993) and excess of protein, which is initially directed towards the formation of muscle tissue, can end up being converted into energy (NRC, 2011; Fracalossi and Cyrino, 2013; Sakomura et al., 2014) accentuating the accumulation of ether extract in the carcass and the imbalance with protein levels.

Thus, it can be considered that the most important relationship in the study was between the percentage of ether extract (fat) in the body composition of the fish, with the gonadosomatic index value. For *Salminus hilarii*, abdominal fat has an inverse relationship with GSI, indicating that it influences the development of gametes (Andrade et al., 2004) and which has also been observed in other fish species (Lal and Singh, 1987). Thus, by observing the development of *A. lacustris* males in this experiment, it was possible to notice that animals with a higher concentration of ether extract in the body had lower gonadosomatic indices, indicating little energy mobilization for gonadal development, and that the accumulation of fat made it difficult to achieve adequate gonadal development.

According to Porto-Foresti et al. (2010), sexual maturity of *A. lacustris* is reached at the age of 4 months; however, the animals in the present study were able to reproduce from the beginning of the experiment (2 months), as demonstrated by the histological analyses carried out, indicating a possible precocity of the study animals, or due to environmental conditions and, in this specific case, a high average temperature, which leads to an increase in the speed of the fish's reproductive physiology.

Fish from the recirculation system had a higher percentage of protein in their body composition; therefore, a higher percentage of muscle tissue, when compared to animals from biofloc technology. According to Guimarães and Adell (1995), most proteins in the body are located in the muscles and connective tissues, constituting 16-22% of muscle mass, resulting in greater protein conversion efficiency in fish under a recirculation system. Animal growth was similar in both systems, RAS and BFT, but BFT animals had a higher percentage of ether extract in their body composition, with high IHS and lower IGS; therefore, it is possible to consider that for the creation of A. lacustris in the BFT system can use lower feeding rates and/or with feed that contains a lower percentage of crude protein; it is worth highlighting that feed is equivalent to 60 to 70% of the cost of fish production and protein is the most expensive item in the composition of the diet, which would be a way to reduce production costs (Silva and Massago, 2019; Brasil, 2019).

Regarding the semen quality observed in this experiment, the best results were observed in RAS fish, which may be associated with a more balanced diet that provides a lower percentage of ether extract in the body composition of the fish. Although fish accumulate fat to begin the reproduction process (Vazzoler, 1996; Costa et al., 2005; Teletchea et al., 2009), to meet the demand for ATPs necessary to support sperm hyperactivity (Cosson et al., 2008), which would make fat accumulation a good indicator and, the excess, can be harmful to reproductive capacity (Donelson et al., 2010; Hayden et al., 2018), which could explain the observed results. Therefore, a possible explanation for the BFT animals having lower values for some important seminal characteristics, when compared to the RAS fish, is that they had a higher body fat rate.

The higher percentage of fast sperm in animals exposed to the systems for three months, with a higher value of progressive motility and higher frequency of flagellar beating in animals in the RAS, may indicate better seminal quality. After one year, RAS animals also showed higher values for important variables linked to seminal quality, such as motility and curvilinear speed.

Higher rates of progressive motility are related to a higher fertility rate in fish, according to a study by Gallego et al. (2013) and Layek et al. (2016), one of the characteristics that guarantees greater fertilization efficiency is progressive motility, and the speed of sperm has a strong connection with high fertilization rates (Rurangwa et al., 2004; Gallego et al., 2013). BCF is a parameter with few bibliographical references linking it to better seminal quality; however, data indicate that sperm speed can be influenced by this factor (Gil et al., 2009; López et al., 2015; Neumann et al., 2017). Seminal quality is directly linked to the fertilization capacity of oocytes, and the quality of male gametes is influenced by nutrition, physiology, well-being, genetics, and environmental factors (Estay et al., 1994; Valdebenito et al., 2015; Zimba et al., 2017).

The information cited above corroborates the findings of this study, since the highest percentage of fast sperm was from the semen of fish exposed to the RAS system for three months, which presented a higher value for the flagellar beat frequency index, a higher percentage of fast sperm, and a higher value of progressive motility, which may indicate better seminal quality.

Motility and curvilinear velocity parameters reflect the physiological condition of the sperm (Peña and Linde-Forsberg, 2000; Kowalski and Cejko, 2019) and sperm motility is generally indicated as one of the most important factors related to the success of fertilization; however, it is important to conduct more detailed assessments of sperm movement correlated with fertilizing capacity are carried out (Liu et al., 1991; Cox et al., 2006). Curvilinear speed is related to better fertilization rates, considering that sperms with a trajectory with more circular movements generally find it easier to locate the oocyte micropyle (Di Chiacchio et al., 2017; Leite et al., 2018). The linearity coefficient, straightness coefficient, average oscillation of the spatial trajectory of the sperm, amplitude of lateral movement of the head, and crossed flagellar beat are rarely used to determine seminal quality in fish and have few reference values (Neumann et al., 2017).

For the animals' seminal quality data, the values of the seminal parameters of animals raised in a recirculation system and biofloc technology found in this study were within levels found in studies with A. lacustris (Yasui et al., 2015; Nascimento et al., 2017; Zimba et al., 2017; Carneiro-Leite et al., 2020).

## Conclusion

It can be concluded that the species *Astyanax lacustris* adapted to both the RAS and BFT systems, presenting similar zootechnical gains in the two cultivation systems tested. In view of the data obtained, it can be concluded that animals from both systems presented adequate growth and reproductive development, but with a slight improvement in seminal quality for males kept in the RAS system.

## Data Availability

The data produced in this project will be deposited in our databases and available to the reader after the publication of the article.
